# Structural and Thermal Characteristics of Buriti Tree Gum (*Mauritia flexuosa*)

**DOI:** 10.3390/polym15071662

**Published:** 2023-03-27

**Authors:** Diego Aires da Silva, Davi do Socorro Barros Brasil, Edinaldo José de Sousa Cunha, Giselle Cristine Melo Aires, Renato Araújo da Costa, José de Arimatéia Rodrigues do Rego, Rosinelson da Silva Pena

**Affiliations:** 1 Food Technology Department, Natural Science and Technology Center (CCNT), University of Pará State (UEPA), Belém 66050-540, PA, Brazil; 2 Chemical Engineering Faculty (FEQ), Institute of Technology (ITEC), Federal University of Pará (UFPA), Belém 66075-110, PA, Brazil; 3 Graduate Program in Food Science and Technology (PPGCTA), Institute of Technology (ITEC), Federal University of Pará (UFPA), Belém 66075-110, PA, Brazil

**Keywords:** hydrocolloids, *Mauritia flexuosa*, polysaccharides, spectroscopy, thermal analysis

## Abstract

A polysaccharide was isolated from the exudate of a buriti tree trunk (*Mauritia flexuosa*). The molecular structure, thermal stability, morphology, crystallinity, and elemental composition of the product were investigated through spectroscopic techniques, such as Fourier-transform infrared spectroscopy (FTIR), nuclear magnetic resonance (NMR ^1^H and ^13^C), and energy-dispersive X-ray spectroscopy (EDS); thermogravimetric analysis (TG), differential scanning calorimetry (DSC), scanning electron microscopy (SEM), and X-ray diffraction (XRD). In addition to NMR molecular modeling studies, were performed to confirm the ^1^H and ^13^C chemical shifts to Gal and Xyl conformers. Buriti tree gum (BG) is an arabinogalactan, containing Rha, Ara, Xyl, and Gal, and degrades almost completely (98.5%) at 550 °C and has a maximum degradation peak at 291.97 °C, with a mass loss of 56.33%. In the temperature range of 255–290 °C, the energy involved in the BG degradation process was approximately 17 J/g. DSC indicated a glass transition temperature of 27.2 °C for BG, which had an irregular and heterogeneous morphology, with smooth or crumbling scaly regions, demonstrating the amorphous nature of BG that was confirmed by the XRD standard. EDS revealed the presence of carbon and oxygen, as well as calcium, magnesium, aluminum, silicon, chlorine, and potassium, in the BG composition.

## 1. Introduction

The Brazilian Amazon has one of the largest reservoirs of plant biodiversity on the planet, composed of a large natural collection of fruits, roots, and countless plant species, with great food, pharmaceutical, therapeutic, and chemical potentials [1]. On the other hand, many natural products used by Brazilian industries are still imported from other countries. An example is gum arabic, the main representative of the segment of natural gums used industrially [2]. It is extracted from the trunk of the *Senegalese acacia* tree [3]. By 2018, the polysaccharides market had raised more than USD 12 billion worldwide, while an annual growth forecast of 4.8% is expected by 2026. The food industry is largely responsible for the consumption of this plant-source material, which is produced by microbiological fermentation [4].

The buriti (*Mauritia flexuosa* L.), which belongs to the Arecaceae family, is an abundant palm in the mesoregion of Baixo Tocantins, in the State of Pará-Brazil, and is very important for economic extraction and subsistence, due to the use of its fruits and leaves [5]. The buriti trunk produces a gum exudate, which protects the palm from threats and microbial attacks [2,6,7]. Materials such as this contain polysaccharides with complex molecular structures, whose origin, size, types of chemical bonds, number of branches and side chains, and spatial conformation govern interactions with water molecules and other molecules [7]. These characteristics confer the physical, chemical, and functional properties of the gums, which define the use of the product [8].

Gums from plant exudates can be used in several applications, such as in confectionery products, salad dressings, frozen and dehydrated foods, the development of systems for the controlled release of drugs, in tablets, adhesives, and glues, and as perfume fixers and repellents, among other applications [9,10,11,12]. According to recent research, polysaccharides from plants have been standing out in several industrial sectors. Arabinogalactans, for instance, have been used in the formulation of biodegradable films, individually [13] or combined with other organic compounds [14,15,16,17]. In addition, these polysaccharides have been used in the medicinal field [18,19,20].

Silva et al. [6] investigated the rheological and colloidal properties of aqueous dispersions of buriti gum, which presented a Newtonian behavior in low concentrations (4–5% *w*/*v*) and a tendency toward a pseudoplastic behavior, in concentrations of 8 and 10% *w*/*v* of gum. The dispersions showed intermediate colloidal stability at pH < 4.0 and greater dispersibility in water at higher pH values. The authors observed that, even at higher concentrations (8–10%), the viscosity of the gum dispersion decreased with increasing temperature, which was attributed to the weakening of the intra- and intermolecular attraction forces of the gum.

The results obtained by Silva et al. [6] for buriti gum already help us understand and identify applications for the product; however, explaining the molecular structure and information on the thermal stability of the gum is important to guide its technological use. Furthermore, understanding the molecular structure of this gum is important for designing studies involving interaction with other compounds as well as improving the properties of products that allow multiple applications, such as the sulfation of arabinogalactans for medicinal use [21,22,23].

The possibilities of use for BG may increase interest in exudates from buriti tree trunks and encourage the management of this plant species, despite the challenges of obtaining the exudate from plant trunks [24]. Therefore, in addition to fruits and other buriti parts already used, BG could become another generator of sustainable products from this Amazonian species.

For these reasons, this paper aims to identify the main functional groups and structural components of BG through spectroscopic techniques such as FTIR and NMR ^1^H and ^13^C; evaluate the thermal behavior of the gum through thermogravimetric analysis and differential scanning calorimetry as well as morphology, scanning electron microscopy, and dispersive energy spectroscopy; and evaluate its crystallinity using X-ray diffraction. Finally, NMR molecular modeling was used to confirm the ^1^H and ^13^C chemical shifts to Gal and Xyl conformers.

## 2. Materials and Methods

### 2.1. Gum Extraction

The exudate found on the surface of the buriti tree trunks was removed using a spatula. Approximately 1 kg of exudate was collected in Cameta (Pará, Brazil) (2°09′02.77″ S, 49°26′15.57″ W) and packed in polyethylene bags at room temperature (≈27 °C). BG extraction followed the methodology presented by Silva and Pena [7]. The exudate was suspended in distilled water (1:10, *w*/*v*) for 24 h at 25 °C, without stirring, to dissolve the soluble fraction. Then, the suspension was filtered through a 1 mm mesh, and the filtrate was centrifuged at 2778× *g* for 10 min (Suprafug 22, Heraeus Sepatech, Minden, Germany). The supernatant received a 1:3 absolute ethanol proportion (solution: ethanol, *v*/*v*). The precipitate obtained (gum) was dried at 50 °C in an air-circulating oven (Quimis Q317M72, São Paulo, Brazil) for 24 h, crushed in an analytical mill (Quimis Q298A21, São Paulo, Brazil), and packed in amber glass, hermetically sealed. The extracted BG was then stored at 25 °C until the analysis took place.

### 2.2. BG Infrared Spectroscopy

BG samples were kept at 25 °C, 75 °C, 100 °C, and 150 °C for 3 h, in an oven with air circulation, to check the effect of the temperature on the decomposition of the polysaccharide component of BG. After being cooled in a desiccator with silica gel, the samples were placed in Eppendorf tubes. Then, the BG samples underwent a spectroscopy analysis in the infrared region on a spectrometer (IR100 spectrometer, Thermo Electron Corporation, Madison, WI, USA). The absorption spectra were obtained in the wavelength range from 400 to 4000 cm^−1^, with a resolution of 2 cm^−1^ and a 64 cm^−1^ scan [25].

### 2.3. NMR ^1^H and ^13^C BG

For NMR ^1^H and ^13^C, 40 mg of BG were dissolved in 4 mL of D_2_O. TSP (3-(trimethylsilyl)propionic-2,2-3,3-d_4_ acid sodium salt) was used as an internal standard and the analyses were performed on a nuclear magnetic resonance spectrometer (Bruker AIII 500MHz, Billerica, MA, USA). Chemical changes were expressed in ppm (δ) [8].

### 2.4. BG Thermogravimetric Analysis

BG mass loss was evaluated through thermogravimetry (TG) and differential thermal analysis (DTA) in a Shimadzu thermobalance (DTG 60H, Shimadzu Analytical, and Measuring Instruments, Kyoto, Japan), with the following conditions: heating ratio of 10 °C/min, temperature range of 25–600 °C, and nitrogen atmosphere with a flow of 10 mL/min. Aluminum crucibles were used as sample holders. For the acquisition and processing of data, the OriginPro 8.0 application (Origin Lab Corporation, Microcal, Northampton, MA, USA) was used [26].

### 2.5. BG Differential Scanning Calorimetry (DSC)

DSC curves were obtained using equipment (DSC Q10, TA instruments, New Castle, USA) operating in the temperature range of −70–200 °C, in three stages (heating–cooling–reheating), and the −10–550 °C range, with a single heating stage. In both cases, a dynamic nitrogen atmosphere was used, with a flow of 50 mL/min, and a heating rate of 10 °C/min. Aluminum crucibles were used as sample holders [26].

### 2.6. Scanning Electron Microscopy (SEM) and Energy-Dispersive Spectroscopy (EDS)

SEM analyses were performed using a scanning electron microscope model LEO-1430, with an EDS Sirius-SD detector attached. The analysis conditions were as follows: electron beam current of 90 µA, constant acceleration voltage of 10 kV, and a working distance of 15 mm. To visualize the BG granule morphology with greater accuracy, a TESCAN scanning electron microscope, VEGA3 model, was used [27].

### 2.7. X-ray Diffraction

The X-ray diffraction analysis (XRD) was performed on a diffractometer, X’PERT PRO MPD from PANalytical. Ceramic X-ray tubes and Cu anodes (Kα1 = 1.540598 Å) were used, with a long fine focus (2200 W-60 kV) and Kβ nickel filter. The analyses were performed under the following conditions: scanning from 5° to 100° in 2θ; voltage of 40 kV; 40 mA current; step size of 0.02° in 2θ and 90 s time/step; divergence 1/4° slit and 1/2° anti spatter; 10 mm mask [28].

### 2.8. NMR Molecular Modeling Studies

A hypothetical molecular structure containing monomers of β-D-Galp and β-D-Xylp was designed and optimized using the DFT method at the B3LYP/cc-pVDZ level, by adjusting such molecule to a minimum local energy conformation. The minimum energy structures are guaranteed by the absence of any imaginary frequency. With all optimized molecules, the theoretical ^13^C- and ^1^H-NMR data were obtained by gauge-independent atomic orbital (GIAO) method [29] by applying the B3LYP/cc-pVDZ level to all atoms. To determine the TMS (internal standard) shielding constants, the same protocol used for the hypothetical molecular structure was strictly followed. All calculations were performed using the software Gaussian 16 [30]. This study was important for eliminating conformational doubts when comparing the experimental NMR data with those described in the literature for Gal and Xyl.

## 3. Results and Discussion

### 3.1. Gum Extraction

The buriti tree gum (BG) was isolated by using a conventional extraction method. The exudate was solubilized in distilled water at room temperature (≈25 °C) while the precipitation of the polysaccharide was performed with absolute ethanol, since the BG was not soluble in ethanol and acetone. The BG extraction yield was 5.4% while the recovery of *Moringa oleifera* Lam. gum was 4% using a conventional extraction process [31]. Although conventional extraction methods present lower yields compared with nonconventional methods, they have the advantage of causing less damage to the structure of polysaccharides, minimizing the loss of bioactivity in the gum [32,33].

### 3.2. BG Infrared Spectroscopy

**Figure 1:**
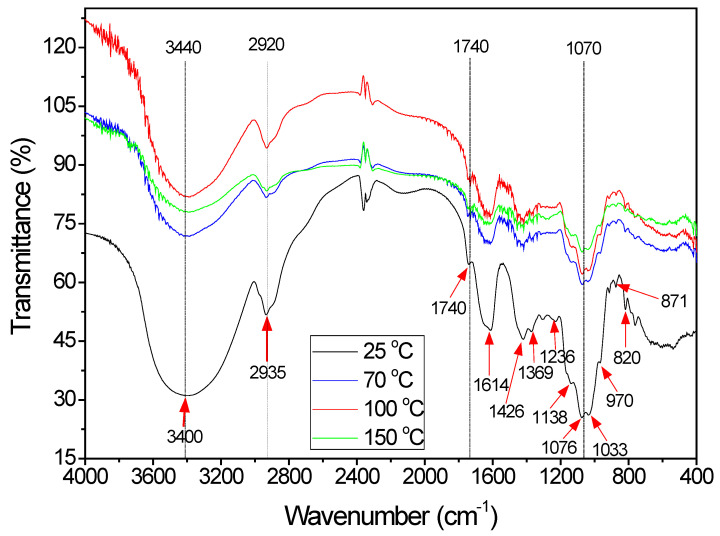
Spectra in the infrared region for BG.

The FT-IR spectrum obtained for BG is shown in Figure 1. The broad and intense band between 3600 and 3200 cm^−1^ is attributed to the stretching vibrations of the O–H group [34]. The band in the absorption range of 2935 cm^−1^ is related to the symmetrical and asymmetrical vibrations of the CH_3_ and CH_2_ groups [35]. The peaks at 1614 cm^−1^ and 1426 cm^−1^ are attributed to the vibrations of asymmetrical and symmetrical stretching of COO– groups, respectively [36], while the peak at 1369 cm^−1^ is associated with vibrations of –CH_3_ and –CH_2_ connections. The 1740 cm^−1^, 1369 cm^−1^, and 1236 cm^−1^ ranges are attributed to the axial deformation of the ester groups C=O, –C–CH_3_, and –C–O–, respectively [37].

The range between 1200 cm^−1^ and 800 cm^−1^ represents the polysaccharide fingerprint. The absorptions in the 1033 cm^−1^ and 1236 cm^−1^ ranges are related to the presence of uronic acids and o-acetyl groups in the molecule [38]. Additionally, the 1033 cm^−1^ range is attributed to the vibration of C–N groups [39] and may be related to the presence of proteins in BG. Baum et al. [25] registered that the 1033 cm^−1^ and 1138 cm^−1^ ranges are attributed to the axial deformation of C–O and may be related to the presence of galacturonan, linked to the main chain of the molecule, while the 1076 cm^−1^ range indicates the presence of neutral sugars, such as arabinose, galactose, and xylose. The presence of xylose can also be related to the presence of 970 cm^−1^ and 1138 cm^−1^ ranges (axial deformation of C–O) [40,41]. The 820 cm^−1^ band refers to the stretching of the anomeric conformations of the α-D-galactopyranose polysaccharides [42]. The reduction in some ranges with the increase in the temperature was evidenced in the 3440, 2920, 1740, and 1070 cm^−1^ wavelengths. According to Liu et al. [43], these losses can be associated with the release of oxygen present in the functional groups of the molecule. Thus, these events can be linked to small mass losses at the studied temperatures, together with the release of water.

### 3.3. Hydrogen Magnetic Resonance Experimental Analysis and Molecular Modeling

**Figure 2:**
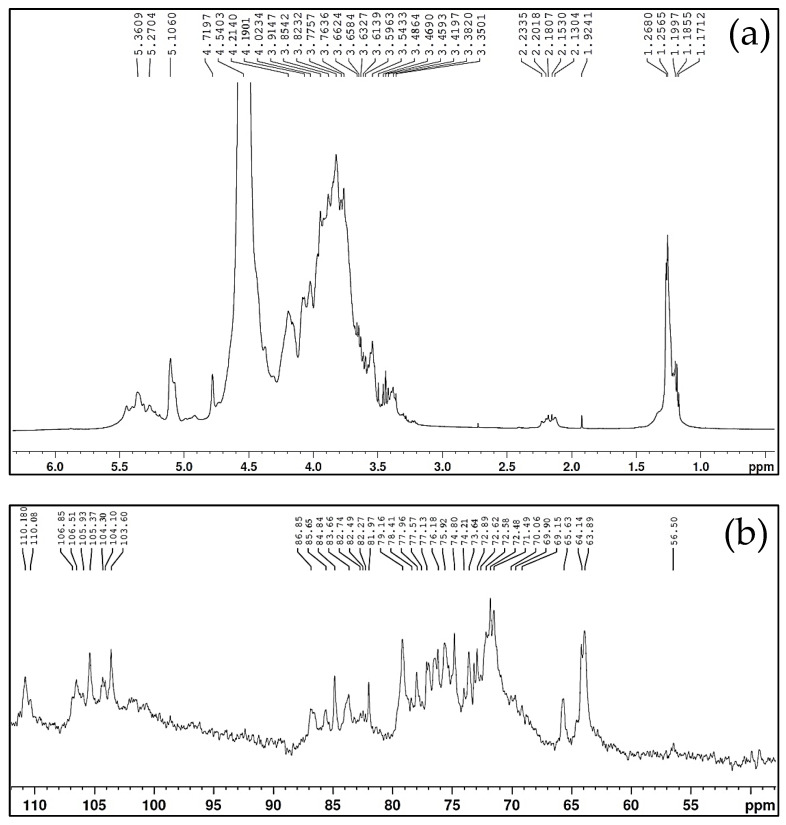
NMR spectra (**a**) ^1^H and (**b**) ^13^C of BG.

When comparing the ^1^H and ^13^C signals from the BG residues in the NMR experimental spectroscopy (Figure 2) with the NMR data in the literature, α- or β- anomeric configuration information is evidenced, and the binding pattern, composition, and sequence of the monosaccharides present in BG are proposed. In the ^1^H NMR experimental spectrum of BG (Figure 2a), signals 4.54, 4.71, and 5.27 ppm are attributed to anomeric protons of β-D-Galp, and residues of β-D-Xylp and α-L-Araf, respectively [44,45]. These values were confirmed in the molecular modeling studies; the chemical shifts obtained to anomeric protons were β-D-Galp (3.88 ppm) and β-D-Xylp (4.69). The values obtained are compatible with those observed in the experimental NMR, where the chemical shifts of β-D-Xylp anomeric hydrogen were greater than those of β-D-Galp. Thus, we can prove that the experimental data, the literature data, and the data obtained by molecular modeling corroborate each other.

In the ^13^C NMR spectrum of BG, the C_1_ resonance of β-D-Galp residues was observed at 104.3 ppm [45,46]. The 103.6 ppm signal is attributed to the anomeric carbon of β-D-Xylp [45,47]. The presence of anomeric signals 105 and 110 ppm indicates that there are Ara residues, with resonance in the furanose form [45,46]. Delgobo et al. [48] related the 5.27/110 ppm H_1_/C_1_ signals to 3-O-substituted α-Araf residues and the 5.27/110.1 ppm signals to bonds (1-3)-α-Araf-(1→3. Sims and Furneaux [49] attributed signals 5.25/110 ppm H_1_/C_1_ to α-L-Araf residues.

The values corresponding to the chemical shifts of signals 4.21/85.6 ppm of H_4_/C_4_, attributed to →3)-α-L-Araf- residues (1→; 3.65/73.6 ppm of H_3_/C_3_ attributed to →6)-β-D-Galp-(1→ residues; 3.76/72.5 ppm H_2_/C_2_, 3.85/82.7 ppm H_3_/C_3_ and 3.91/74.2 ppm H_5_/C_5_ attributed to →3, 6)-β-D-Galp-(1→ residues; 4.54/104.3 ppm H_1_/C_1_, 3.77/72.6 ppm H_2_/C_2_, and 4.19/69.9 ppm H_4_/C_4_ attributed to →3)-β-D-Galp-(1→ residues; 3.48/77.1 ppm of H_3_/C_3_ and 3.61/71.4 ppm of H_4_/C_4_ attributed to residues of β-D-Xylp- (1→; 3.76/71.4 ppm of H_3_/C_3_ and 3.45/72.4 ppm of H_4_/C_4_ attributed to α-L-Rhap-(1→ residues; 3.46/75.9 ppm H_3_/C_3_ and 3.77/77.1 ppm H_5_/C_5_ attributed to β-D-GlcpA-(1→ residues were found in the NMR spectra for BG and compared with the results presented by Molaei and Jahanbin [45]. These results suggest that BG is an arabinogalactan with the presence of Rha, Ara, Xyl, Gal, and galacturonic acid.

To certify the comparison previously made between experimental ^13^C NMR data and the literature data, the results obtained by molecular modeling for the chemical shifts of the main carbon atoms of β-D-Galp and β-D-Xylp residues were compared with those assigned experimentally (Table 1). Residual analysis showed that differences between chemical shifts ranged from 0.12 to 3.85 ppm with a mean of 2.26 ppm. These values obtained are within the expected range for the use of the molecular modeling tool to confirm the structure of organic compounds, as observed in the literature [50,51]. In this way, we can verify that the structural analysis using NMR for the structure of BG is reliable.

### 3.4. BG Thermogravimetric Analysis

**Figure 3:**
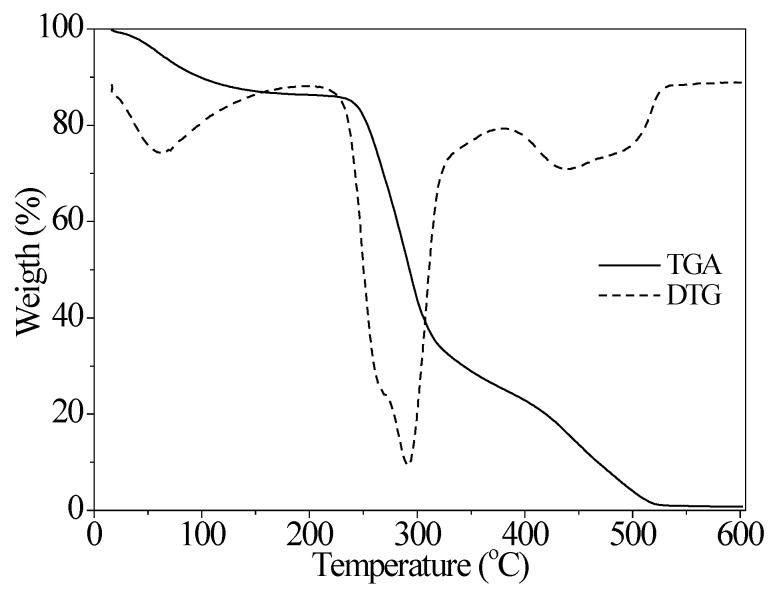
Thermogravimetric curves of BG.

The BG TGA curve (Figure 3) presented three stages of degradation, with a profile similar to that observed for other polysaccharides, such as gum arabic [52], guar gum [53], xanthan gum [54], and cashew gum [26]. The maximum mass loss observed was 98.52% at 527 °C. The first mass loss stage, in the 42.22 °C–110.11 °C range, was attributed to water evaporation and involved a 17.51% mass loss, with a maximum degradation temperature of 74.83 °C. The second stage occurred in the 259.75 °C–312.91 °C range and involved the greatest mass loss (56.33%), which was attributed to the degradation of the polysaccharide molecular chain [52]. For this stage, the maximum degradation temperature was 291.97 °C. The third and last stage of degradation occurred in the 402.23 °C–488.34 °C range and was attributed to complete polysaccharide decomposition [26,55]. In this stage, a 24.68% mass loss was observed, with a maximum degradation temperature of 433.69 °C.

Arabinogalactans from gum arabic showed a thermogravimetric peak at 315 °C, which was attributed to the maximum degradation temperature of the gum [56]. According to Al-Maqtari et al. [57], arabinogalactans show moderate temperatures of degradation. This behavior was also reported by Guan and Zhong [58], who observed the thermal degradation of arabinogalactans from gum arabic used as a wall material in the encapsulation process. These results suggest that BG is more suitable to be used in applications that do not involve high temperatures.

### 3.5. BG Differential Scanning Calorimetry

**Figure 4:**
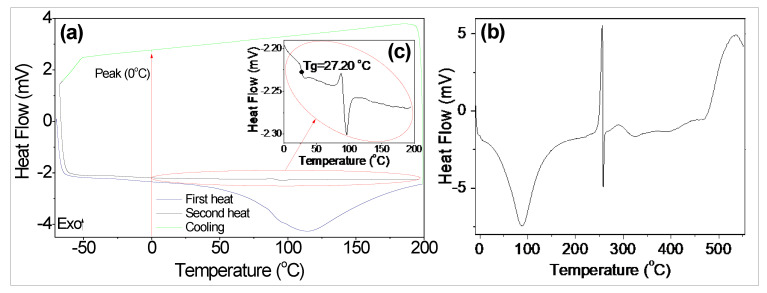
DSC of BG. (**a**) Heating–cooling–heating cycles; (**b**) DSC of the gum for the temperature range of −10–550 °C; and (**c**) glass transition temperature (Tg).

The DSC thermograms obtained for BG with 11% of moisture content on a dry basis (db), for a heating–cooling–reheating cycle, in the temperature range of −70–200 °C, are shown in Figure 4a. The thermograms define a thermal history for the polysaccharide that is part of BG, which did not undergo thermal degradation up to 200 °C. This behavior corroborates the thermogram in Figure 3, where the thermal degradation of the polysaccharide only occurs at a temperature above 250 °C. Figure 4b, in turn, shows the DSC thermogram for heating BG in the temperature range from −10 °C to 550 °C. The initial, peak, and final temperatures, as well as the enthalpies involved in the different events, are shown in Table 2.

The first heating stage (Figure 4a) resulted in a large endothermic peak (Table 2), which can be attributed to the loss of free water from the polysaccharide [59]. During cooling, the heat capacity decreased monotonically with evidence of ice formation, due to the presence of a thin peak at 0 °C, which can be attributed to the presence of residual free water in the molecule [60].

The BG glass transition temperature (Tg), obtained using DSC, was 27.20 °C (Figure 4c). López-Franco et al. [61] and López-Franco et al. [62] found Tg values of 33.88 °C for mesquite gum (*Prosopis* spp.) with 5.9% moisture content (bs) and 33.54 °C for mesquite gum (*Prosopis pallida*) with 2.6% moisture content (bs). Chouaibi et al. [63] observed Tg values of 39.8 °C for guar gum with 6.4% moisture content (bs) and 40.5 °C for Retama gum with 3.7% moisture content (bs), while Chaires-Martínez et al. [64] found 58.57 °C Tg for locust bean gum with 3% moisture content (bs).

Tg is the temperature at which a material undergoes the transformation from the gummy to the vitreous state, which is closely related to the amount of water in the gum, the gum’s ability to gain (adsorb) water, and the temperature [65]. Thus, the lower Tg value observed for BG can be attributed to its higher moisture content, when compared with the moisture content of the other gums mentioned [66].

After the Tg event, an endothermic peak was observed at 96.48 °C, with enthalpy (ΔH) of −49.16 J/g, in the second heating curve (Figure 4c, Table 2). The value of ΔH for this event was reduced by 88.6% when compared with the value of ΔH observed for the endothermic peak of the first heating stage. This endothermic event can be attributed to the enthalpic relaxation of BG, due to energy gain, by increasing mobility for Tg [67].

In the DSC of Figure 4b, in addition to the endothermic event at 88.02 °C, with a ΔH −417.07 J/g (Table 2) attributed to the water evaporation of BG, three other events were observed, at temperatures above 200 °C. The second event, exothermic and with a temperature peak (T_p_) of 255.4 °C, was followed by an endothermic event, with a T_p_ very close to the previous one (257.58 °C), and, later, by another exothermic event, with a T_p_ at 290.59 °C. The enthalpies involved in these events were approximately the same magnitude (Table 2) and can be attributed to the degradation of the polymer that is part of BG, due to the breaking of bonds between molecules [68]. Mothé and De Freitas [26] observed endothermic events at 245 °C and 360 °C for cashew gum, in an air atmosphere, with ΔH values of 142.2 and 26 J/g, respectively. The authors attributed these events to polysaccharide degradation, as well as other exothermic events related to the oxidation of carbon residues formed during the decomposition of cashew gum.

In the FT-IR spectrum (Figure 1), ranges close to 2400 cm^−1^ are related to the release of CO_2_ [26]. In addition, the decrease in groups such as –OH (3400 cm^−1^), –CH_3_ (2935 cm^−1^) and –CH_2_, and ester groups (1740 cm^−1^) with increasing temperature is a sign that these groups are involved in oxidation and rupture reactions, at temperature levels that promote BG degradation. Similar results were observed for xanthan gum and methyl cellulose [54]. Mothé and De Freitas [26] observed that in a nitrogen atmosphere, the degradation of the polysaccharide promoted the release of CO, with the degradation occurring practically in the same ranges observed for BG, which was attributed to the formation of aldehydes and alkanes due to decomposition. Thus, the presence of CO_2_ and CO suggests the breakdown of the glycosidic bonds of the polysaccharide [43].

### 3.6. Characterization of the Microstructure and XRD Analysis

**Figure 5:**
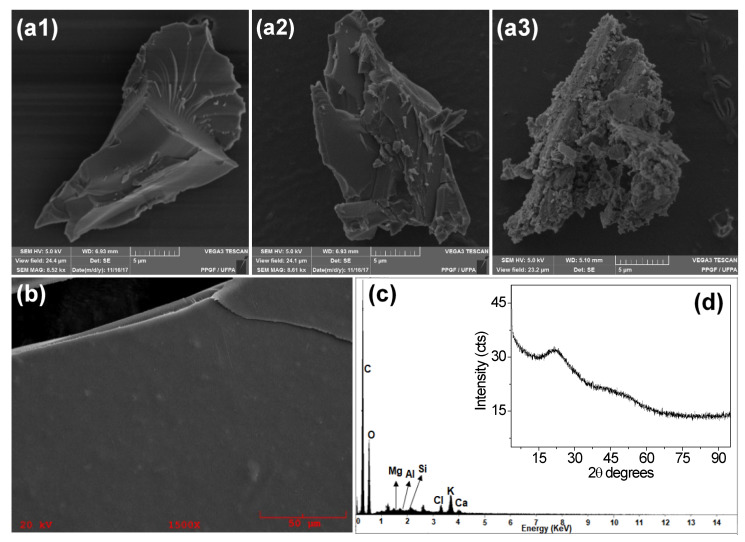
SEM images of (**a**) powdered BG (**b**) after drying at 50 °C for 24 h; (**c**) EDS spectrum and (**d**) XRD of the gum.

There is major variability among the microstructures of gums from different origins, but all have irregularities in their granular structures [69]. SEM images of BG powder (Figure 5a1–a3) also show the presence of very distinct and irregular structures, indicating that the BG granules have a heterogeneous morphology, with scaly and smooth regions (Figure 5a2), or even crumbling and scaly regions (Figure 5a3), similar to gum karaya, a natural gum obtained from the exudate of *Sterculia urens* [27,70].

During the drying process for BG (50 °C/24 h), a film was formed, which was crushed to obtain BG powder. The micrograph of the film (Figure 5b) showed a flat, homogeneous surface, with small roughness in some points and the presence of some cracks in the structure. These characteristics suggest that BG can be used in film production [71].

The irregularity of the BG microstructure, in turn, indicates the amorphous nature of the product, which is confirmed by the BG XRD standard (Figure 5d), which presented a small peak at 2θ = 21.34°, followed by a broad plateau without peaks, a characteristic of amorphous materials. This pattern is similar to that observed for almond gum, gum arabic, and arabinogalactan [72,73].

The energy-dispersive spectroscopy (EDS) analysis, shown in Figure 5c, reveals the presence of carbon and oxygen, originating from the polysaccharide carbon chain, as well as calcium, magnesium, aluminum, silicon, chlorine, and potassium. The presence and affinity of these elements, for water or other molecules, could be fundamental for the knowledge of stability and for defining the technological uses for BG [74].

Elucidating the structure of BG can amplify the opportunities for new research aimed at the utilization of this polysaccharide in several industrial fields. One example is the use of arabinogalactans in the production of composites that are used in the development of biodegradable packaging films [13], individually or combined with carboxymethyl cellulose, methylcellulose, polyvinyl alcohol, or even nanocomposites [14,15,16,17]. Additionally, knowledge of the molecular structure of this polysaccharide and its thermal stability can assist in the development of promising materials from the BG. In the medicinal field, arabinogalactans display various health benefits, such as antitussive activity, antibody response to the pneumonia vaccine, acting as the basis for drug delivery systems, the inhibition of liver metastasis, and analgesic and anti-inflammatory properties [18,19,20].

## 4. Conclusions

The gum obtained from the exudate of the buriti tree trunk was characterized. The yield extraction of polysaccharides was 5.4%. Infrared spectroscopic data indicated that BG is an arabinogalactan, containing Rha, Ara, Xyl, and Gal as well as galacturonic acid. The BG glass transition temperature was set at 27.20 °C and the maximum temperature of the greatest mass loss was 291.97 °C, attributed to the degradation of the molecule through the oxidation of the carbon residues formed. DSC revealed endo- and exothermic events between 250.5 °C and 305.87 °C, involving energies of 17.30–22.19 J/g, attributed to the loss of CO_2_ and CO due to decomposition through the rupture of glycosidic polysaccharide bonds. SEM indicated that BG can be used for the production of biofilms and XRD indicated that BG is a strictly amorphous polysaccharide at room temperature. Buriti tree gum has structural and physical properties similar to gums from other plant sources that are commercially used. Its similarity might encourage further studies on this polysaccharide from this Amazonian palm tree. This is the first report on the structural and thermal characteristics of BG, and the results are important for supporting studies relating to the application of BG in the food, chemical, or pharmaceutical industries.

## Figures and Tables

**Table 1:** Comparison between theoretical and experimental ^13^C NMR chemical shifts (ppm) for β-D-Galp and β-D-Xylp residues.

Position	Experimental	Theoretical	Residue Module
anomeric carbon (β-D-Xylp)	103.6	100.07	3.53
anomeric carbon (β-D-Galp)	104.3	104.42	0.12
C_3_ to →6)-β-D-Galp-(1→	73.3	76.79	3.49
C_2_ to →3, 6)-β-D-Galp-(1→	72.5	74.34	1.84
C_5_ to→3, 6)-β-D-Galp-(1→	74.2	72.91	1.29
C_2_ to →3)-β-D-Galp-(1→	72.6	74.67	2.07
C_4_ to →3)-β-D-Galp-(1→	69.9	69.01	0.89
C_3_ β-D-Xylp- (1→	77.1	73.25	3.85
C_4_ β-D-Xylp- (1→	71.4	68.80	2.60
C_3_ to β-D-GlcpA-(1→	75.9	76.79	0.89
C_5_ to β-D-GlcpA-(1→	77.1	78.80	1.70

**Table 2:** Temperatures and changes in enthalpy associated with the heating and cooling stages of buriti tree gum.

Event	Temperature (°C)			∆H (J/g)
	Initial	Peak	Final	
Heat stage				
First heating stage	51.66	114.35	176.55	−431.52
Second heating stage	87.00	96.48	108.69	−49.16
Degradation				
Event 1	43.83	88.02	132.20	−417.07
Event 2	250.50	255.14	255.98	17.30
Event 3	256.78	257.58	259.77	−17.74
Event 4	257.33	290.59	305.87	22.19

∆H: Enthalpy variation.

## Data Availability

The data are contained within the article.
